# Is What Low-Income Brazilians Are Eating in Popular Restaurants Contributing to Promote Their Health?

**DOI:** 10.3390/nu10040414

**Published:** 2018-03-27

**Authors:** Alinne de Paula Carrijo, Raquel Braz Assunção Botelho, Rita de Cássia Coelho de Almeida Akutsu, Renata Puppin Zandonadi

**Affiliations:** Research Group in Nutritional and Nourishment Quality, Department of Nutrition, University of Brasilia, Brasilia DF 70910-900, Brazil; alinnecarrijo@yahoo.com.br (A.d.P.C.); raquelbabotelho@gmail.com (R.B.A.B.); rita.akutsu@gmail.com (R.d.C.C.d.A.A.); renatapz@yahoo.com.br (R.P.Z.)

**Keywords:** low-income population, popular restaurant, lunch, energy density, nutrition

## Abstract

This study evaluates the healthfulness of the meals offered to and consumed by low-income Brazilians in Popular Restaurants (PR). It is a cross-sectional, exploratory study. The final sample includes 36 PRs, respecting the stratification criteria for each of the five Brazilian regions. To identify the quantity and quality of food consumption, consumers’ meals are evaluated. The sample calculation uses a minimum of 41 consumers in each PR. Consumption evaluation is carried out by weighing and direct observation of the meal that each consumer served to his plate. Each dish of the meals had its Technical preparation files (TPF) developed by observing the production and weighing all the ingredients. Evaluations of Energy density (ED), meal’s weight components and sodium composition are conducted. Plate’s composition is compared to “My plate” guidelines United States Department of Agriculture (USDA). The final sample includes 1771 low-income Brazilians consumers. The plate of PRs consumers is adequate only for the “protein group” in comparison to “My plate”. Rice and beans compose more than 50% of the plate’s weight, as expected, since it is a Brazilian habit of consumption at lunch. Thus, grains are the major group consumed by PRs consumers. The average ED for all PRs is 1.34 kcal/g. Regarding sodium content, rice and main courses presented the highest values and are classified as high, according to Food and Drug Administration (FDA). Concerning sodium, PRs are putting Brazilian low-income population at risk for chronic diseases. However, in general, PRs are good choices because they promote access to cheap and quality traditional Brazilian foods.

## 1. Introduction

Popular Restaurants (PR) is an assistance program created by the Brazilian Government and it is characterized as food service units that offer inexpensive and healthy meals to low-income population in Brazil [1,2]. The purpose of this program is to guarantee the social rights of feeding, consolidated by the Universal Declaration of Human Rights [3]. It is also to improve health of low-income population, since food access and choices have a substantial impact on prevention and treatment of several diseases [4,5,6].

In the last decades, globalization and modernization have been changing feeding habits. A consumption increase of fatty and sugary industrialized foods, with high energy density (ED) and low fiber content can be verified. Further, people have no time to prepare meals and they are frequently eating out. In Brazil, eating out represents, to low-income population, an access to snacks that are cheap, easy and fast to intake. Usually, they have inadequate nutrients and poor sanitary conditions [2,7,8]. This situation results an increase of chronic and foodborne diseases that are very common in low-income population in developing countries such as Brazil [4,5,6]. Besides this panorama, we have many people that do not have access to food, or to good quality meals to guarantee survival and health.

Despite the need of eating out and knowing the difficulties to access healthy food, the Brazilian government provides more than 120,000 daily meals through the PRs. In this plan, PRs have to offer access to healthy and cheap meals as well as improve cultural food habits to low-income population [2].

However, there is no report about evaluation of this social program as a tool to promote health and to guarantee food access to low-income population in the literature. Then, the aim of this study is to evaluate the healthfulness of the meals offered to and consumed by low-income Brazilians in Popular Restaurants (PR).

## 2. Materials and Methods

This research is transversal and exploratory based on direct documentation. The Research Ethics Committee (Protocol No. 0372/10) approved it.

### 2.1. Sampling

To select PRs, we used the following inclusion criteria: (i) a food service belonging to the Popular Restaurants program of the Brazilian Federal Government; (ii) signature of the Institutional Acknowledgement Agreement by the dietitian responsible for the food service; (iii) open during lunch; and (iv) service of more than 500 meals daily. Given the inclusion criteria, 65 PRs were eligible to be part of the study. From the selected population (*N*), the sampling plan was calculated considering an error (e) of a daily meal and a level of significance (α) of 5% [9]. A simple random sample was estimated through the procedure “survey select” of the SAS 9.1.3 program. The final sample included 36 PRs, respecting the stratification criteria for each of the five Brazilian geographical regions.

To identify the quantity and quality of food consumption, consumers’ meals were evaluated. A minimum sample of 41 consumers in each PR was necessary. The inclusion criteria were: be a frequent consumer (more than 3 times a week) and be over 18 years old. The excluding group from the sample was pregnant women because of different nutritional needs. The individuals’ selection occurred while they were waiting in line to serve their meal at lunchtime (self-service). Invitations to participate occurred to the first person in line, then the 15th person, and this pattern was used until sample was completed. Participants signed the acknowledgement and agreement term and evaluation occurred during 4 days at the PRs. On the first day, participants were recruited and, on the other 3 days, their meals were weighed and evaluated. After recruitment, the same participant continued evaluation for the rest of the study. For this study, participants had to complete the three days of consumption evaluation to be qualified. Social demographic variables, sex and age groups (<21 years old, 21–30, 31–40, 41–50, 51–59 and ≥60 years old) were used to qualify the analyzed variables of this study.

The analyzed variables were: (i) the weight of the meal (g) of each component of the meal, e.g., main course (protein preparation offered on the menu, usually of animal origin and decisive for the selection of other items), garnish (menu item accompanying the main course, which may have as main ingredients vegetables, pasta, tubers, and cassava flour dishes), side dishes (items such as rice and beans, culturally daily consumed by the Brazilian population), salad and dessert (fruit or sweets) [10]; (ii) the portion of each component on the plate; (iii) the consumption of fruit and vegetables; (iv) the composition of garnish (pasta or vegetable) and desserts (sweets or fruits); and (v) the energy density of the meal.

### 2.2. Consumption Evaluation

The consumption evaluation was carried out by weighing and direct observation of the meal that each consumer served in his or her plate, according to the procedures proposed by Savio, Costa, Miazaki and Schmitz [11]. The meal weight was measured to relate the percentage of each dish preparation in the meal composition. It was important to check the meal weight on a scale to evaluate whether the observed portions of each dish were correctly described.

To evaluate the nutritional composition of the meals, we developed technical preparation files (TPF) according to the protocol proposed by Camargo and Botelho [12]. Based on the data recorded in the TPFs, the nutritional value was calculated using the information available in the Brazilian Food Composition Table [13]. When this information did not exist, we used scientific publications and labels on processed food products and then entered the information into the system database (DietWin^®^, Porto Alegre, Brazil).

The nutritional analysis was carried out considering the Total Energy Value (TEV) (2000 kcal/day) [1] as a benchmark, following the Brazilian guideline recommendation. The Brazilian Surveillance agency uses 2000 kcal as the energy parameter for nutrition labeling in the Brazilian products. We considered that the lunch meal should provide 40% of TEV [1]. After the calculation of the energy values of each preparation in 100 g of food, we calculated ED expressed in kcal/g of food. According to the Centers for Disease Control and Prevention [14], the preparations were classified as: high energy density (4 to 9 kcal/g), medium energy density (1.5 to 4 kcal/g), low energy density (0.7 to 1.5 kcal/g) and very low energy density (0 to 0.6 kcal/g). The results of these analyses helped to evaluate if the PR program is promoting access to quality food and health.

For sodium evaluation, we compared the results to the Food and Drug Administration standards for sodium content of foods [15]. They considered a general rule of 5% daily value (DV) or less of sodium per serving is low; 20% DV or more is high. Additionally, FDA settles that 140 mg of sodium or less per serving is “low sodium”; 35 mg of sodium or less per serving is “very low sodium”.

In some PRs, Salt sachets were available to consumers who could add salt to their plate before consumption. This salt addition was not measured, and this could be a possible bias of this research. Therefore, sodium results might be underestimated.

### 2.3. Statistical Analysis

Descriptive analysis was carried out by SPSS 20^®^ software. The amount of dietary preparations’ intake (in grams) and the consumption of fruits and vegetables were adjusted considering intrapersonal variability to estimate the usual consumption of individuals through MSM^®^ software (2012).

T-test was used for comparisons between sexes and ANOVA for comparisons among age group and sexes. It was established that *p* < 0.05 was statistically significant.

## 3. Results and Discussions

The final sample included 1771 low-income Brazilians consumers (60% male and 40% female; age range 45 ± 17.39 years) distributed proportionally among the PRs of the five regions of Brazil.

Healthy eating is a mix of many factors, including stage of life, preference, access to food, culture, and traditions. The PRs were created to promote access to food respecting habits and meal quality. In Brazil, lunch is the main meal and PRs are open for it from 11:00 a.m. to 2:00 p.m. to guarantee access to a greater number of consumers. Menu is the same from the time the PR opens until it closes.

According to Brazilians food habits, PR menus should include the following preparations: rice (grains), beans, main course (protein), garnish (pasta, some cooked vegetables or other carbohydrate dishes such as couscous and “farofa” (manioc flour dish)), salad and dessert [2]. It is important to highlight that all PRs offer a fixed meal composed of one protein dish (main course), one garnish, one type of rice, one type of beans, a mixed salad, and one type of dessert. Therefore, consumers only have one menu option each day they eat at the PR. Energetic value and sodium intake are related to the mean portions consumed by the population of the whole study. PRs are located in different regions, but since they are part of a governmental program, all of them have to offer an equal fixed number of dishes. They may vary the type of protein, garnish, salads and desserts, but not the number of options.

**Figure 1:**
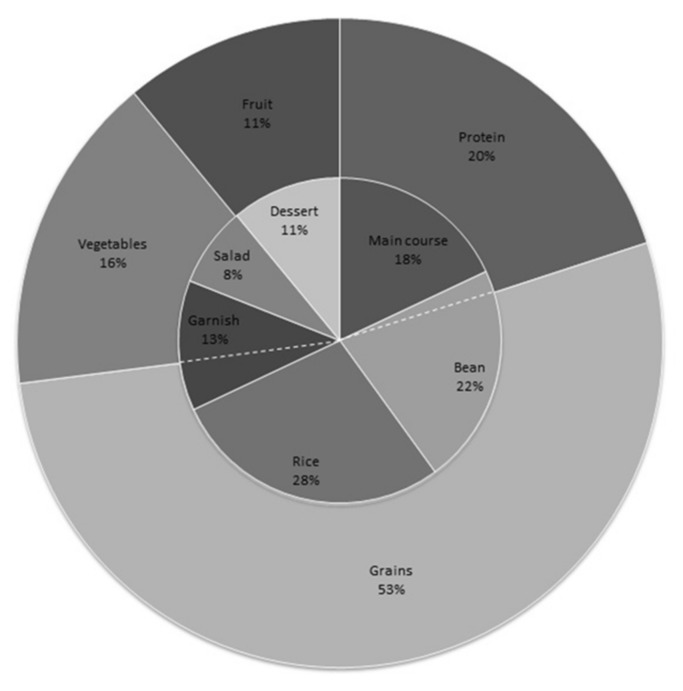
Plate composition by type of preparation consumed by low-income Brazilians in Popular Restaurants and distribution according to recommend groups of “My plate” United States Department of Agriculture (USDA) groups.

Figure 1 represents the composition of the main plate (percentage of weight) consumed by low-income Brazilians in PRs and the distribution according to recommend groups of “My plate” (USDA). According to “My plate”—United States Department of Agriculture (USDA) food guide—the plate should have the composition of 20% protein group; 30% grains group; 30% vegetables group; and 20% fruit. “My plate” illustrates (proportionally in the plate) groups of foods that should be present and consumed for a healthy diet.

The PR consumers’ plate is adequate only for the “protein group” in comparison to “My plate”. In the USDA guide, all foods made from meat, poultry, seafood, beans, peas, eggs, and soy products are in the “protein group”, which recommends 5–6 portions daily. Thus, PRs should provide 2.0–2.5 portions (40%) of this group. In our study, PRs consumers ate about 117.88 g ± 21.76 of the main course (protein group) and 139.67 g ± 48.52 of beans. According to “My plate”, beans are part of the “grains group” and “protein group”. In Brazil, we consume beans with 50% broth and 50% beans, so we have an average amount of 70 g of beans. Considering that beans have about 5% protein (according to the Brazilian composition table) [13], we considered this percentage as part of “protein group” and added the remaining percentage to “grains group”. Therefore, at lunch, low-income Brazilians consumed about 2.5 portions of the protein group. It is important to highlight that protein/meat is the most expensive group of foods in Brazil. Probably, low-income population would not buy these products to eat at home. Thus, the highest consumption of this group at PRs is important to guarantee protein access and to promote a balanced and healthy diet during the day.

Evaluating protein group consumption, there was no statistical difference between males and females (*p* = 0.063). However, there was a significant difference among age groups: females over 60 and less than 21 years old ate less protein than the other age groups (*p* < 0.00), while males over 51 years and younger than 21 years ate less protein (*p* < 0.00).

We highlight that in our study rice and beans comprise more than 50% of the weight of the plate, which was expected since it is a Brazilian habit of consumption at lunch. Thus, grains are the major group (53%) consumed by PRs consumers. It is almost 2× greater than “My plate” recommendation for this group. Comparing to this food guide, people need to consume 6–7 portions of grains (rice is included) daily. In Brazil, we consider that the lunch meal should provide 40% of TEV [1], so we have to guarantee 2.4–2.8 portions of grains (about 230 g) at lunch. In our study, the average rice portion was 181.93 g ± 74.93 (Table 1) and pasta consumption (as a type of garnish) was 79.42 ± 39.35. We also have to include part of the beans’ weight. Therefore, the grains group amount consumed at lunch in PRs was about 330 g, more than the USDA recommendation. Table 1 shows TEV and the average consumption of meals, ED and sodium density (SD) of each preparation that compose the plate (in grams).

The consumption of rice and beans (together) is a Brazilian habit at lunch. Barbosa [16] evaluated Brazilians food habits (2136 participants) and 94% of Brazilian’s meals showed the combination of rice and beans in their study. The amino acids combination of these two groups represents the major part of Brazilian’s protein intake [17].

Despite the recommendation from the Brazilian Food Guide (BFG), [18], we observe that the consumption of rice and beans at home has been declining over time [19]. The explanation could be the reduced time to prepare food at home. Therefore, the PRs are contributing to this consumption within low-income Brazilians with low cost.

The adequate consumption of fruits and vegetables is associated to the reduction of chronic and cardiovascular diseases [20]. According to WHO/FAO, the individual recommendation of fruits and vegetables intake is about 400 g/day. In our study, we verified the consumption of 122.44 g ± 33.04 of fruits and vegetables, which represents about 30% of the daily recommendation, not the expected 40%. In comparison with “My plate”, fruit and vegetables should compose 50% of the graphic representation of the plate, while only 27% of this group were on the PRs plates. Men consumed more salads and vegetables than women (*p* = 0.000; *p* = 0.003). When comparing groups by age, the population over 60 years and the ones younger than 21 are statistically different from the groups between 21 and 40 years (*p* = 0.000), eating less salads.

Corroborating with our data, Levy, Claro, Mondini, Sichieri and Monteiro [21] showed that the availability and the consumption of fruits and vegetables at home in Brazil is under the recommendation, contributing only 2.8% of daily TEV per person. Fruits and vegetables are very perishable and demand frequent shop and adequate storage. Therefore, it is improbable that low-income Brazilians consume large amount of these products outside of PRs to compensate low consumption there. Besides, “My plate” USDA guide recommends the plate’s composition be 50% fruits and vegetables, which does not occur in meals consumed by low-income Brazilians in PRs. In this viewpoint, PRs are not providing all the needs for fruits and vegetables to promote health.

The consumption of fruits (68.05 g ± 47.68) in PRs is 2.5× higher than the offer of sweet desserts (24.40 g ± 30.25). When comparing fruit consumption between male and female, there were statistical differences with higher consumption on the male group (69.18 g, *p* = 0.026). Statistical differences among age groups were not observed (*p* = 0.451, female; *p* = 0.805, male). It is important to highlight that the sweet desserts offered in PRs were made from fruits with sugar (such as guava paste, banana candy paste, and coconut candy). It is a very important data showing that PRs are not providing the total amount of fruits, but they are careful on planning more fruits than sweets for desserts to avoid health damage. Another relevant aspect is that in Brazil we consume higher amounts of fruits for breakfast and snack meals. Since we evaluated only lunch, it is not possible to guarantee that the fruit consumption during the day is under recommendation.

In general, the average energy intake for low-income Brazilians in PRs lunch was 881 kcal (±222.02). There were no statistical differences among sex (*p* = 0.145). It is higher than recommended by the Brazilian Worker Food Program (WFP) of 800 kcal [1] to guarantee adequate energy intake to maintain the organism. If the energy intake was the single parameter to guarantee health, the PRs program would be reaching its goal. However, PRs should guarantee food quality access.

The ratio of the energetic value and the weight of the meal is estimated by ED which is one of the coefficients that increases obesity and overweight or underweight on population, and foods ED affects population health [22].

The average of ED for all PRs, in this study, was 1.34 kcal/g (±0.25). This value is higher than the American Institute for Cancer Research’s [23] recommendation (1.25 kcal/g). The EDs shown in our study were smaller than the EDs presented by Stella [24] (1.98 kcal/g) and Canella [25] (1.43 kcal/g). The first one evaluated the ED consumption of 710 consumers in São Paulo/Brazil. Canella [25] evaluated the ED of menus offered by 21 food service in São Paulo/Brazil. ED varied among the five regions, being higher in the north (1.51 kcal/g ± 0.16) and lower in the south (1.15 kcal/g ± 0.12). Differences were not observed between the center-west region and the southeast region (*p* = 0.701). All other comparisons of ED were statistically different (all *p* values = 0.000).

Energy density as a measurement of overall diet has been the focus of many recent studies. The high-ED foods or meals are known as an “obesity-inducing dietary pattern”. High ED diets are rich in fat and energy, but low in fiber, fruits and vegetables. Moreover, higher ED is inversely related to diet quality, which may encourage weight gain, increase the risk of metabolic syndrome and diabetes [26,27,28].

In this study, 78.1% were low ED (and 21.9% were medium ED). Although most meals presented low ED, the average TEV is in accordance with WFP recommendation. It could be explained by the portions size of the food offered by PRs, especially rice and beans that presented low mean ED. Main courses had higher EDs. These data show that the PRs are offering meals with expected ED adequacy, contributing to prevent overweight, obesity and other chronic diseases associated to high ED food consumption. However, it is important to encourage the population to consume more fruits and vegetables for better micronutrients intake.

The Institute of Medicine has alerted to reduce salty foods [29], and the FDA set standards for the sodium content of foods [15]. They considered a general rule of 5% DV or less of sodium per serving is low; 20% DV or more is high. Additionally, FDA settles that 140 mg of sodium or less per serving is “low sodium”; 35 mg of sodium or less per serving is “very low sodium”.

Regarding sodium content, rice and main courses presented the highest values and according to FDA are high in sodium [15]. Rice presented the highest value because it represents the biggest portion on the plate. Main courses did not represent big portions, but PRs generally use pork sausages, hot dog sausages and jerked beef that are cheap, but present high sodium content. All others are classified as medium Sodium Density, except salads and desserts groups which presented low sodium and very low sodium, respectively. It is important to emphasize that the mean sodium content at lunch meal (considering all the preparations and respective portion of the day) was about 2400 mg. This sodium amount in only one meal represents the total sodium expected for an entire day. Furthermore, the total meal sodium content might be underestimated, since the salt sachet was not considered.

## 4. Conclusions

These data are alarming, and PRs need to review their TPF to lower sodium contribution and highlight the importance of increasing the consumption of vegetables and fruits. It is also important to increase the offer of low ED foods. In some PRs, the sodium intake could be even worse if consumers added extra salt to their plates.

Concerning sodium, PRs are putting Brazilian low-income population at risk for chronic diseases, mainly cardiovascular diseases [29,30]. A better menu planning is necessary in these restaurants to guarantee sodium reduction throughout time, so the population can adjust without rejecting the new preparations. However, in general, the PRs are good choices for low income Brazilians because they promote access to cheap and quality Brazilian traditional foods. Better menu planning to increase fruit and vegetables offer in place of grains group is necessary. Fruits should be the only option for desserts in PRs and educational strategies are necessary to encourage more salad intake. Protein offer is adequate and it is important for this population because of its high cost. However, the negative aspect is that this protein offer is often achieved with high fat and high sodium meat sources. Low cost better option should be encouraged.

PRs contribute to promote health in low-income Brazilians overall (energy intake and macronutrients distribution). This program is a big step to guarantee Brazilians the universal right to food. However, in general, since the purpose of the program is to offer healthy food for low-income population, menu planning should be the focus of the program. PRs are good choices for low-income Brazilians because they promote access to cheap and traditional Brazilian foods, but better protein quality, more vegetable and fruits and lower sodium content are major challenges to this program.

Further studies can be developed to evaluate this population after a longer period of consumption at the PRs. Other variables such as lunch’s contribution on the daily intake and the type of food acquired at home may bring new data to evaluate the program’s importance to these consumers.

## Figures and Tables

**Table 1:** Average and standard deviation (SD) of consumption (grams), total energetic value (TEV) Energy density (ED) and Sodium content of meals and preparations by low-income Brazilians in popular restaurants.

	Average in Brazil	Rice	Bean	Main Course	Garnish	Salad	Dessert
Meal weight ± SD (g)	648.34 ± 133.08	181.93 ± 74.93	139.67 ± 48.52	117.88 ± 21.76	86.56 ± 23.44	51.70 ± 20.47	70.50 ± 37.76
Energetic value ± SD (kcal)	881 ± 222.02	147.93 ± 60.3	108.80 ± 61.20	213.25 ± 92.54	151.85 ± 133.01	35.02 ± 20.94	68.60 ± 46.90
Energy Density ± SD (kcal/g)	1.34 ± 0.25	1.48 ± 0.60	1.09 ± 0.61	2.13 ± 0.93	1.52 ± 1.33	0.35 ± 0.21	0.97 ± 1.32
Sodium content (mg/portion)	2362.87	795.03	445.54	749.72	241.50	118.39	12.69
Sodium Percent Daily Value (% DV)	98.45	33.13	18.56	31.24	10.06	4.93	0.53
